# Sample Size Calculation. Comment on Quantitative Ultrasound and Dual X-Ray Absorptiometry as Indicators of Bone Mineral Density in Young Women and Nutritional Factors Affecting It, *Nutrients*, 2019, *11*, 2336

**DOI:** 10.3390/nu12010019

**Published:** 2019-12-20

**Authors:** Jose M. Moran, Antonio Sanchez Fernandez

**Affiliations:** 1 Metabolic Bone Diseases Research Group, University of Extremadura, 10003 Cáceres, Spain; 2 Servicio de Tocoginecología, Hospital Universitario de Cáceres, 10003 Cáceres, Spain; gineantonio@gmail.com

We have read with interest the recently published paper by Schraders et al. [1] that reports correlations between quantitative ultrasound (QUS) and the gold-standard technique for osteoporosis diagnosis, dual X-ray absorptiometry (DXA), in a group of 54 healthy young women (18–26 years) from Manawatu, New Zealand, and also the relationship with nutrient intake. The manuscript concludes that QUS may provide a reasonable indicator of osteoporosis risk in young women but may not be an appropriate diagnostic tool and also that increased calcium intake is recommended for this group, regardless of bone mineral density (BMD). 

In the materials and methods section, the authors provide detailed information about the sample size calculation that leads to a final sample of 54 healthy young women. We would like to highlight that, as presented, the sample size calculation in this manuscript makes no sense and does not justify the sample size of 54 participants included in the study. It does not imply that the conclusion of Schraders et al. is inaccurate, but the calculation of the sample size is. 

The authors based their calculation of the sample size in having enough statistical power to detect a putative correlation between DXA and QUS following the procedures described by Charan and Biswas [2]. The manuscript of Charan and Biswas does not provide methods for correlation studies but for cross-sectional studies/surveys, clinical trials or clinical interventional studies and animal studies. As the Schraders et al. study could be classified as a cross-sectional study, the sample size should focus on the characteristics of the studied variables (qualitative or quantitative). As the authors indicate that they used the predicted population of 18–25-year-old females (267,100 in New Zealand) in 2017 [3], it necessarily indicates that the authors have based their sample size calculation in a qualitative variable which makes no sense in the study of a putative relation between DXA and QUS. It could make sense if the reference variable for the sample size were “diagnosis of osteoporosis”, but it was not studied in the manuscript. So the authors calculated the sample size assuming a 90% confidence level, a margin of error of 10%, and a variability of 10%. What does this 10% of variability represent? It is a key value in the sample size calculation for qualitative variables, but it should represent a prevalence or a distribution, and again it makes no sense to use it in a correlation study. The sample size was correctly calculated, accounting for potential incomplete data sets/drop-outs with the data provided, but the data provided does not allow the calculation of an accurate sample size for the proposed study design. 

A correlation study needs a threshold probability for rejecting the null hypothesis (type I error rate; usually 5%), the probability of failing to reject the null hypothesis under the alternative hypothesis (type II error rate; usually 80%), and an expected correlation coefficient between the studied variables (DXA and QUS) [4]. Neither the predicted population nor the variability has a value in the sample size calculation for a correlation study. 

We would like to emphasize that an inaccurate sample size calculation does not imply that the conclusion of Schraders et al. is misleading but, as discussed, we think that the sample size of 54 participants is not justified in this study, and hence, it cannot be assured that the study is not underpowered. The inaccuracy described here should have been detected and amended during the peer review process. 
